# Research progress on circRNAs in type 2 CRS

**DOI:** 10.3389/fimmu.2026.1785434

**Published:** 2026-04-22

**Authors:** Zhai Liu, Zhi Chen

**Affiliations:** Department of Otolaryngology Head and Neck Surgery, Changsha Hospital of Traditional Chinese Medicine (Changsha Eighth Hospital), Changsha, Hunan, China

**Keywords:** circRNA, diagnosis and treatment, molecular sponge, post-transcriptional regulation, type 2 chronic rhinosinusitis

## Abstract

Circular RNAs (circRNAs) constitute a recently identified class of non-coding RNAs that are widely distributed in eukaryotes. Characterized by the absence of 5′ caps and 3′ polyadenylated tails, circRNAs may regulate gene expression through multiple mechanisms. Recent studies have shown that certain circRNAs contain binding sites complementary to microRNAs (miRNAs), enabling them to function as molecular sponges that sequester miRNAs and inhibit their suppressive effects on mRNA expression. Emerging evidence indicates that specific circRNAs are involved in the regulation of immune cells and cytokines associated with type 2 chronic rhinosinusitis; however, direct clinical evidence supporting associations between circRNAs and the diagnosis or severity of type 2 chronic rhinosinusitis remains unavailable. In this study, the molecular mechanisms of circRNAs in type 2 inflammation are systematically summarized, established experimental evidence is distinguished from clinical applications that remain to be validated, and a theoretical basis is provided for future research on type 2 chronic rhinosinusitis.

## 1 Introduction

Chronic rhinosinusitis (CRS) is commonly subclassified into type 1, type 2, and type 3 CRS according to the expression profiles of T-cell-associated factors. Among these subtypes, type 2 CRS is typically characterized by marked eosinophilic infiltration of the nasal mucosa, which makes clinical management particularly difficult (1). Although type 2 CRS has been reported predominantly in European and North American populations (2), recent studies have indicated an increasing prevalence of type 2 inflammation in Asian populations (2–4). In addition, frequent comorbidity with allergic rhinitis, asthma, and non-steroidal anti-inflammatory drug-exacerbated respiratory disease (N-ERD) (2, 5, 6) has been associated with a higher risk of postoperative recurrence (7, 8). Accordingly, the identification of more reliable and efficient biomarkers remains an urgent priority in clinical diagnosis and treatment. With Th2 cells serving as a key immunologic link in the pathogenesis of type 2 CRS, circRNAs have been reported to participate in the regulation of Th2 cell differentiation and to be involved in allergic responses (9). Circular RNA (circRNA) is characterized by a covalently closed circular structure and greater resistance to endonuclease-mediated degradation; compared with mRNA, miRNA, and lncRNA, it exhibits greater molecular stability (10, 11).

In current clinical practice, type 2 CRS can be further divided into distinct endotypes according to the degree of tissue eosinophilic infiltration, comorbidities (such as asthma and N-ERD), and inflammatory molecular features. These subtypes exhibit substantial heterogeneity in underlying pathological mechanisms and therapeutic responses. However, stratified analyses based on patient subtypes have not been performed in most existing circRNA studies; therefore, caution is warranted when extending circRNA-related mechanisms to the entire population with type 2 CRS.

Through a comprehensive review of recent literature on type 2 CRS and circRNA, with particular emphasis on studies addressing related immune cells, cytokines, and diagnostic and therapeutic strategies, a series of circRNA molecules implicated in type 2 CRS was identified and compiled. This synthesis is expected to support more in-depth investigation of the molecular basis of type 2 CRS, provide a novel perspective for clinical evaluation of disease severity, and contribute to the refinement of therapeutic approaches for this condition. Notably, current research on circRNA in type 2 CRS has been conducted primarily in animal models and *in vitro* systems; direct clinical evidence supporting its value as a diagnostic biomarker or an indicator of disease severity remains unavailable.

CircRNAs are generated from precursor mRNAs through back-splicing, and their biogenesis is regulated by complementary sequences within flanking introns, RNA-binding proteins, and splicing factors. CircRNA expression profiles exhibit marked tissue specificity across different cell types. Under inflammatory conditions such as type 2 CRS, the enrichment of specific circRNAs may be closely associated with altered splicing factor activity and shifts in cellular composition within the local inflammatory microenvironment. In addition to summarizing mechanistic evidence, this study explicitly differentiates established mechanisms from putative clinical applications that have yet to be validated. The following sections address the intrinsic relationship between circRNA and type 2 CRS from multiple perspectives and conclude with future research directions.

## 2 Research progress on circRNAs in type 2 CRS

A study examining the epidemiological trends of type 2 CRS in China (1) reported that the overall prevalence of CRS in the domestic population ranged from 4.8% to 9.7%. Mixed-type inflammation represents the predominant inflammatory pattern, and the incidence of type 2 CRS has shown a gradual increase in recent years (2, 4). Owing to the high recurrence rate of type 2 CRS across all age groups, patients often experience substantial psychological and physical burdens, markedly reducing quality of life and increasing economic costs (12, 13). From an immunological standpoint, type 2 CRS shares similarities with allergic rhinitis (1), as both are mediated by Th2 cells (14) and involve multiple bioactive molecules, including IL-35 (15), IgE (16), and IL-17 (17), whose dysregulated expression contributes to the onset and progression of type 2 CRS. Nonetheless, the pathogenesis of type 2 CRS remains complex. A substantial body of evidence, both direct and indirect, has indicated that circRNAs may contribute to the pathogenesis of type 2 CRS through regulation of immune cells, such as Th1/Th2 cells and macrophages, as well as modulation of related cytokine expression, including IL-1 and IL-6 (18–22).

### 2.1 Involvement of circRNAs in the regulation of immune cells in type 2 inflammation

**Figure 1:**
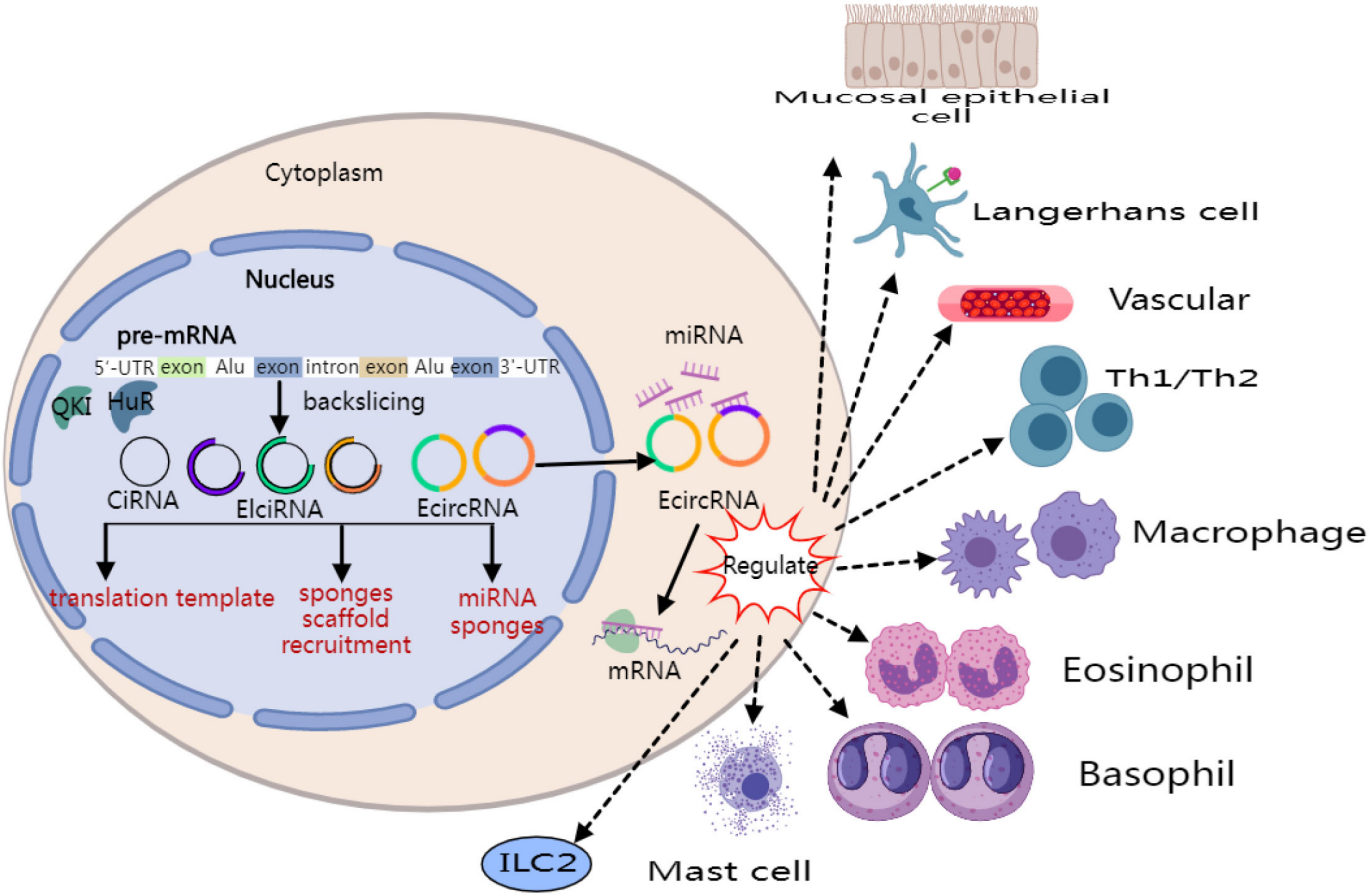
CircRNA-mediated regulation in type 2 CRS.

From an immunopathological perspective, type 2 CRS shares several features with type I hypersensitivity reactions. The major manifestations include Th1/Th2 imbalance, activation of type 2 innate lymphoid cells (ILC2s), dendritic cells, macrophages, and mast cells, and local eosinophil recruitment. Recent studies have indicated that circRNAs may act as molecular sponges by competitively binding miRNAs, thereby modulating the functional states of the aforementioned immune cells; this effect appears particularly relevant to the regulation of cells involved in type I hypersensitivity reactions (23, 24) (**Figure 1**). For example, circRNAs may affect the differentiation trajectory of helper T cells through sequestration of specific miRNAs (9), suggesting a potential regulatory role within the immune network of type 2 CRS.

During mast cell activation, miR-221–222 has been shown to be significantly upregulated (25). Although Bazan et al. reported an association between circR-284 and miR-221 in the serum of patients with carotid plaque rupture, the role of this regulatory axis in type 2 CRS or type I hypersensitivity reactions has not yet been investigated. This gap in the literature indicates that the expression profile and immunoregulatory relevance of the circR-284/miR-221 axis in patients with type 2 CRS should be systematically examined in future studies.

As principal antigen-presenting cells, dendritic cells are integral to the initiation and persistence of type 2 inflammation. Using a murine cardiac transplantation model, Chen et al. demonstrated that circSnx5 decreased dendritic cell immunogenicity and thereby prolonged graft survival through regulation of the miR-544/SOCS1 axis (26). In that study, graft survival time and histopathological evaluation were used as the primary endpoints, and circSnx5-modified dendritic cells markedly suppressed recipient T-cell activation by inducing an immune-tolerant phenotype. Although type 2 CRS was not directly examined, mechanistic evidence for circRNA-mediated immune regulation was provided. If a comparable immunomodulatory approach could be achieved in the local nasal mucosa, Th2-type immune responses might theoretically be attenuated, thereby offering a potential conceptual basis for anti-allergic immune intervention in type 2 CRS. However, the applicability, safety, and delivery of this strategy in a chronic inflammatory setting remain to be further validated.

Macrophages, particularly M2 macrophages, have been shown to infiltrate the tissues of eosinophilic rhinosinusitis with nasal polyps to a significant extent; moreover, eosinophil recruitment to inflamed areas of the nasal cavity is promoted through the secretion of chemokines such as CCL13 (27, 28). At present, several studies have preliminarily characterized the roles of circRNAs in macrophage polarization and the regulation of type 2 inflammation (29, 30); however, the available evidence remains largely correlational and lacks rigorous functional validation and detailed mechanistic analysis. In addition, Zhang et al. reported in a mouse model of allergic rhinitis that circCramp1l expression was upregulated and that it participated in inflammatory responses through modulation of the miR-532-3p/HMGB1/Drp1 axis (23); another study suggested that circDdx17 may also be involved in immune regulation via miR-17-5p. Nevertheless, systematic clinical cohort data remain lacking to determine whether the expression levels of the aforementioned circRNAs in peripheral blood or nasal mucosal tissues are associated with disease severity, inflammatory phenotype, or treatment response in type 2 CRS. Accordingly, the current evidence supporting circRNAs as tools for immunophenotyping or as therapeutic targets in type 2 CRS remains limited, and rigorous validation through studies integrating clinical samples with functional experiments is still needed.

Notably, current functional studies of circRNAs in type 2 CRS have focused predominantly on the molecular sponge mechanism; however, the functions of circRNAs are not confined to this mode of action. CircRNAs could exert scaffold-like effects through interactions with RNA-binding proteins, function as protein decoys, and even serve as translational templates for the production of functional polypeptides. Nevertheless, these alternative mechanisms have not yet been systematically described in the context of type 2 CRS. Thus, current understanding of the role of circRNAs in type 2 CRS remains constrained by a relatively narrow mechanistic framework, and future studies should pursue comprehensive validation across multiple functional dimensions.

### 2.2 CircRNAs participate in the regulation of type 2 inflammatory cytokines

Type 2 CRS is centered on Th2 cells, which produce a range of cytokines that drive type 2 inflammation, including IL-4, IL-5, IL-13, and IL-6. Previous studies have shown that circRNAs can regulate cytokine production in allergic disease models through binding to miRNAs (23). MiRNAs are non-coding, single-stranded RNA molecules approximately 22–25 nucleotides in length that participate in the regulation of human protein synthesis and cellular function through modulation of mRNA (31). A study by Luan et al. (32) confirmed that MicroRNA-21-5p was significantly upregulated in nasal mucosa with type 2 inflammation and aggravated type 2 inflammation in the nasal mucosa of patients with CRS with nasal polyps by targeting the Glucagon-like peptide-1 receptor/IL-33 signaling pathway. Mimmi et al. (33) reported that miR-25-3p and miR-185-5p contributed to the progression of type 2 CRS through effects on cytokine-related pathways, including the IL-4/13 receptor. In addition, miR-21 can affect the progression of inflammation in the nasal mucosa by suppressing PTEN expression in rhinosinusitis-affected tissue (34). Because circRNAs contain sequences complementary to those of miRNAs, corresponding miRNAs can be sequestered, thereby inhibiting miRNA-mRNA interactions and modulating translation (35).

In recent years, with the rapid development of genomics and transcriptomics, circRNAs have become widely recognized as molecular sponges for miRNAs, thereby modulating the expression of downstream genes. Through this mode of interaction, a circRNA/miRNA/mRNA regulatory network is formed, which in turn influences disease progression; notably, this role has been supported by direct experimental evidence in studies of type 2 CRS (36).

In experiments using calcitonin gene-related peptide (CGRP)-induced macrophages, activation of the IL-6-associated inflammatory axis was observed (37, 38). CircRNA_007893 was shown to suppress miR-485-5p expression through complementary base pairing, which subsequently resulted in a marked increase in IL-6 levels and the induction of an inflammatory response (30). In another study (39), a murine model of nasal allergy was established, and elevated Circ_0067835 expression was detected in mucosal tissue (p < 0.001, n = 6). Silencing Circ_0067835 reduced the levels of type 2 cytokines (IL-4, IL-5, IL-9, and IL-13) and ILC2s in the murine model. In addition, Circ_0067835 was found to target and positively regulate miR-155 expression, while GATA-3, a downstream target of miR-155, was also regulated by the Circ_0067835/miR-155 axis. CircRNA regulatory axes do not operate as isolated linear pathways; instead, they frequently converge to form complex regulatory networks. For example, circRNA_404013 expression (p < 0.001, n = 79) was significantly increased in the peripheral blood of patients with allergic rhinitis, and this circRNA may contribute to the pathogenesis of type 2 inflammation by modulating brain-derived neurotrophic factor expression via miR-182-5p (40). CircRNA_404013 has therefore been proposed as a potential candidate for preventive strategies targeting type 2 CRS; however, the underlying mechanisms require further investigation. In immune cells, circPan3 enhances IL-13 stability, thereby prolonging the immune response (41). As a representative cytokine in type 2 CRS, IL-13 induces effector B-cell differentiation and the rapid synthesis and release of IgE, while also promoting inflammatory cascades and nasal mucosal secretion. CircARRDC3 (n=20) negatively regulated miR-375 expression; after circARRDC3 knockdown, the resulting miR-375 overexpression inhibited both IL-13-induced eosinophil chemokine expression and cellular apoptosis. Accordingly, circARRDC3 appears to be involved in type 2 CRS and provides further insight into the pathogenesis of type 2 inflammation (42).

In addition to the aforementioned cytokines, Th2 cells produce the lineage-specific transcription factor GATA3. GATA3 is involved in chromatin remodeling during Th2-cell activation and transcription, thereby promoting IgE release as well as the activation and recruitment of downstream effector cells (43). Zhu et al. (9) reported that circHIPK3 upregulated the expression of GATA3, a transcription factor in Th2 cells, thereby contributing to the development of type 2 inflammation or worsening patient symptoms. On the basis of these observations, regulation of circHIPK3 to reduce IgE expression in the nasal mucosa, with the aim of alleviating or treating inflammation, may represent a potentially valuable direction for future investigation that is distinct from current IgE-targeted monoclonal antibody therapies.

In nasal allergic reactions, IL-1β expression is significantly upregulated and directly correlated with symptom severity, indicating its positive biologic activity in these processes (44). Another study reported that in mouse articular chondrocytes pretreated with IL-1β, comprehensive profiling identified more than one hundred circRNAs with increased expression, including circRNA-30365 and circRNA-36866, along with a substantial number of downregulated circRNAs (45). Thus, it may be hypothesized that circRNAs function as molecular sponges through the circRNA/miRNA/mRNA axis to regulate the expression of cytokines such as IL-1β, thereby participating in the pathogenesis and progression of type 2 inflammation or other nasal allergic disorders; however, the precise mechanisms involved remain to be elucidated.

## 3 Role of circRNAs in the diagnosis and treatment of type 2 CRS

The diagnosis of type 2 CRS is based primarily on symptom assessment, specialized clinical examination, and laboratory testing. Among these approaches, histopathological examination of nasal mucosal tissue is regarded as the gold standard for diagnosis; however, it is an invasive procedure. For patients with suspected type 2 CRS, the identification of noninvasive diagnostic criteria with high predictive value before surgery is of substantial importance for preoperative treatment planning and prediction of postoperative therapeutic outcomes. To date, no clinical studies have conclusively established circRNAs as diagnostic biomarkers for type 2 CRS. In patients complicated by type 2 inflammatory diseases, such as asthma or allergic rhinitis, CT findings showing abnormal soft-tissue density in the olfactory cleft on the medial side of the middle turbinate, or identifying the lateral ethmoid sinus as the primary site of inflammation, may to some extent suggest the presence of type 2 CRS (46–49).

A study of bronchial asthma reported that periostin (POSTN) secretion in mucosal exudates was upregulated in response to type 2 inflammatory stimuli, including Th2 cells, IL-4, and IL-13. Secreted periostin may in turn act on fibroblasts and epithelial cells, thereby inducing airway epithelial remodeling (50, 51). Because allergic rhinitis belongs to the same disease spectrum as asthma, studies of allergic rhinitis have likewise confirmed that periostin levels in nasal secretions are correlated with disease severity (52) and are also associated with IL-4 and IL-13 levels. In addition, detection of specific IgE (sIgE) in nasal secretions (53) highlights the importance of investigating local inflammatory responses within the nasal cavity and further suggests that analysis of nasal secretions may serve as a preliminary approach for assessing the severity of allergic rhinitis, representing an important direction for the future development of noninvasive diagnostic strategies for type 2 inflammatory diseases involving the nasal mucosa. Previous studies have demonstrated a close association between circRNAs and type 2 CRS (**Table 1**); however, no published reports have been identified regarding circRNA expression in nasal secretions during type 2 inflammation or its correlation with the severity of allergic reactions. Given their intrinsic stability, circRNAs may have substantial potential as auxiliary diagnostic markers for type 2 inflammation, particularly in the inherently unstable environment of nasal secretions, and therefore warrant further investigation. Although the closed-loop structure of circRNAs confers strong resistance to nuclease degradation *in vitro*, their actual stability in the complex milieu of nasal secretions, which is characterized by high protease activity and abundant microbial flora, has not yet been systematically evaluated. In addition, pre-analytical variables, including sample collection methods, storage temperature, and RNA extraction protocols, may substantially affect circRNA detection results; at present, no standardized procedures have been established. Thus, the application of circRNAs as noninvasive biomarkers in nasal secretions remains at an exploratory stage.

**Table 1:** Expression and functions of circRNAs in type 2 CRS.

CircRNA	Target	Expression pattern	Potential effects	Source
circSnx5 (26)	miR-544	Low expression	Regulates dendritic cell function	Experimental
circCramp1l (23)	miR-532-3p	High expression	Activates Th2 cells, epithelial injury	Experimental
circ_0067835 (39)	miR-155	High expression	Upregulates type 2 cytokines and type 2 innate lymphoid cells	Experimental
circRNA_404013 (40)	micRNA-182-5p	High expression	Promotes inflammation	Experimental
circARRDC3 (42)	miR-375/KLF4	High expression	Eosinophil chemokine expression, cell apoptosis	Experimental
circRNA-HIPK3 (9)	miRNA-495	High expression	Th2 cell differentiation	Experimental
circ_0099188 (29)	miR-381-3p/PPP3CA	High expression	Macrophage polarization, promotes inflammation	Experimental
circRNA-0000520 (36)	miR-556-5p/NLRP3	High expression	Promotes inflammation, cell pyroptosis	Experimental
circRNA-ARF3 (58)	miR-205-5p/SIRT5	Low expression	Relieves inflammation, inhibits cell apoptosis	Experimental

Most of the aforementioned studies are conducted in animal models or *in vitro* cell experiments; moreover, some of the cited studies do not explicitly report quantitative parameters such as fold change, P values, or sample size.

In addition, various circRNAs have been identified in the intestinal mucosa (54, 55). Preliminary work by our group has demonstrated an association between the gut microbiota and type 2 inflammation in the nasal mucosa (56); however, whether circRNAs participate in the immunoregulatory axis linking the gut and the nasal cavity remains unclear. Further studies are needed to determine whether gut-derived circRNAs can serve as indirect indicators of type 2 inflammatory status in the nasal cavity.

At present, circRNA detection relies mainly on RNA sequencing, real-time quantitative PCR (qPCR), digital PCR, and fluorescence *in situ* hybridization (FISH). RNA-seq is suitable for global profiling, although its sensitivity is influenced by library construction strategies. qPCR is widely used for quantitative validation; however, specific primers spanning back-splice junctions must be designed to avoid interference from linear RNAs. Digital PCR enables absolute quantification and is particularly suitable for low-abundance samples. FISH allows localization of circRNA expression within tissues, although its throughput is limited. In the clinical translation of type 2 CRS, the sensitivity, reproducibility, and standardization of detection methods remain major issues that require prompt resolution.

In recent years, immunotherapeutic regimens, primarily involving monoclonal antibodies targeting IL-4Rα, IL-5, and IgE, have been widely used in patients with type 2 CRS, particularly in those with failed surgical treatment. Although favorable outcomes are often achieved, some patients are considered to have responded poorly after six months of therapy, resulting in treatment discontinuation. Accordingly, alternative therapeutic strategies need to be identified, and further investigation in inflammatory cell biology, molecular immunology, and genetics is required to achieve a more comprehensive understanding of the molecular and genetic basis of type 2 CRS.

Numerous *in vitro* and animal studies have confirmed that miRNAs play an essential role in type 2 inflammation and possess distinct therapeutic relevance. For example, SNHG16 upregulates leukemia inhibitory factor expression by binding to miR-106b-5p, thereby enhancing JAK1/STAT3 signaling and promoting the progression of type 2 inflammation (57). A noteworthy study by Zheng’s team further illustrated the unique relevance of circRNAs: in mouse nasal mucosal epithelial cells treated with IL-4/IL-13, marked injury characteristic of type 2 inflammation was observed; notably, circARF3 overexpression ameliorated histopathological abnormalities, apoptosis, and mucosal inflammatory responses in the nasal tissues of these mice (58). This observation may provide a useful basis for the development of novel therapeutic strategies. In addition, Wang et al. (42) reported that in the mucosa of patients with type 2 CRS, circRNA arrestin domain containing 3 (circARRDC3) expression was positively correlated with that of Krüppel-like factor 4 (KLF4). CircARRDC3 was shown to promote the progression of nasal mucosal inflammation through modulation of the miR-375/KLF4 axis. This study provides persuasive evidence for the clinical relevance of circRNAs in the treatment of type 2 CRS and suggests their potential to attenuate disease-related tissue injury. Meanwhile, an animal study showed that therapeutic intervention with miR-135a effectively reduced eosinophil and mast cell infiltration in the nasal mucosa of mice and restored the Th1/Th2 balance toward Th1 dominance (59).

## 4 Prospects for circRNAs in type 2 CRS

Although numerous studies have examined the therapeutic application of miRNAs in allergic rhinitis, their inherently single-stranded structure renders them relatively vulnerable to exonuclease-mediated degradation (60, 61), creating challenges for stability and preservation. By contrast, circRNAs can act as competing endogenous RNAs (ceRNAs) that bind to miRNAs and modulate gene expression (62). In addition, the covalently closed-loop structure of circRNAs confers greater stability (63); accordingly, circRNAs may assume a distinct role in the pathogenesis of type 2 CRS. CircRNA-based therapeutic strategies have already been explored in several neoplastic diseases (64, 65). Although the application of circRNAs to the treatment of type 2 CRS remains at an early stage, current evidence suggests that future research in this field may be directed toward the following aspects: 1. Clinical cohort validation: Prospective cohorts should be established to clarify the associations of specific circRNA expression levels (e.g., circ_0067835 and circARRDC3) in nasal mucosal tissue and peripheral blood with the endotypes, treatment responses, and prognostic outcomes of type 2 CRS. 2. In-depth mechanistic investigation: In primary cell cultures or humanized animal models of type 2 CRS, it should be determined whether circRNAs participate in Th2 immune regulation through non-sponging mechanisms, such as protein binding or translation. 3. Standardization of noninvasive detection: Multicenter studies should be conducted to establish standardized protocols for the collection, storage, and detection of circRNAs in nasal secretions and to evaluate their feasibility as noninvasive auxiliary diagnostic markers. 4. Validation of therapeutic targets: Delivery systems, such as lipid nanoparticles or exosomes, should be applied in animal models to investigate the therapeutic efficacy and safety of circRNA overexpression or interference for local inflammation in type 2 CRS. 5. Investigation of the gut-nose axis: By integrating fecal microbiota data with fecal circRNA profiles, preliminary studies should be performed to determine whether gut-derived circRNAs participate in the systemic regulation of nasal type 2 inflammation.

## 5 Conclusion

The preceding sections summarize the characteristics and biological functions of circRNAs, together with studies examining their associations with immune cells, cytokines, and the diagnosis and treatment of type 2 CRS. A novel perspective is thereby provided for the clinical evaluation of type 2 CRS, with the potential to improve disease management; however, research specifically focused on type 2 CRS remains at an early stage. As an area within non-coding RNA research, this field still requires extensive investigation. It is anticipated that future studies of circRNAs will lead to more effective therapeutic strategies and more reliable prognostic outcomes for patients with type 2 CRS, ultimately improving quality of life.
